# Toward a fully coherent tender and hard X-ray free-electron laser via cascaded EEHG in fourth-generation synchrotron light sources

**DOI:** 10.1107/S1600577523006586

**Published:** 2023-08-24

**Authors:** X. Yang, L. H. Yu, V. Smaluk, T. Shaftan, X. Huang

**Affiliations:** a National Synchrotron Light Source II, Brookhaven National Laboratory, Upton, NY 11973, USA; b SLAC National Accelerator Laboratory, Menlo Park, CA 94025, USA; RIKEN SPring-8 Center, Japan

**Keywords:** storage-ring-based free-electron lasers, cascaded echo-enabled harmonic generation, diffraction-limited synchrotron light sources, coherent radiation, SASE and XFEL oscillators

## Abstract

Free-electron-laser-based beamlines utilize fully coherent laser pulses with extremely narrow bandwidth allowing direct use of X-rays without monochromators. This could be very beneficial for all users of current and future fourth-generation diffraction-limited synchrotron light sources (DL-SLSs) who need narrowband full-coherence high-brightness X-ray pulses. Based on our previous finding, *i.e.* that separating the two stages of echo-enabled harmonic generation (EEHG) with a few extra bending-magnet sections provides an effective way to increase the momentum compaction of chicane 1, one can simultaneously achieve adequate prebunching at extremely high harmonics as well as keep the energy modulation to the ideal minimum. This could open the door for cascaded EEHG, toward fully coherent tender and hard X-ray wavelengths. Built on our compact design of a twin-pulse seeding electron beam with an adjustable delay and timing jitter at the level of a few femtoseconds, a cascaded EEHG can be implemented, which includes two EEHG beamlines, where the radiation pulse generated by the first beamline with harmonic *h*_1_ could be used as the input seed laser pulse to the second beamline with harmonic *h*_2_. Hence, the second radiator could potentially reach very high harmonics [*h* = *h*_1_(20)*h*_2_(25–100)] from 500 to 2000, corresponding to tender and hard X-ray wavelengths. It is demonstrated that the cascaded EEHG scheme is compatible with almost any current or planned fourth-generation DL-SLS, with significant benefits for space-limited storage rings in particular. The main advantage is that this scheme requires almost no change of the storage-ring lattice and is fully compatible with other beamlines. Current proposals for rings with much longer straight sections would add self-amplified spontaneous emission as another viable option for storage-ring-based free-electron lasers.

## Abstract 2

Built on our compact design of a twin-pulse seeding electron beam with an adjustable delay and a few femtoseconds timing jitter, a storage-ring-based cascaded EEHG can be implemented, which consists of two EEHG beamlines, where the radiation pulse generated by the first beamline could be used as the input seed laser pulse to the second beamline. Hence, the second radiator could potentially reach very high harmonics, toward fully coherent tender and hard X-ray wavelengths.

## 1. Introduction

Synchrotron light sources (SLSs) are major tools for a wide range of scientific endeavors, in particular due to the high pulse repetition rate they enable. However, even for recent fourth-generation SLSs, there still exist several key constraints: the electron bunches are long (at least a few picoseconds) and have a large energy spread (of the order of 10^−3^) and low peak current (<300 A) compared with linac sources. Because of these limitations, some form of external seeding from a laser is required to produce shorter coherent radiation (CR) pulses before one can take advantage of the ultra-stable (on the level of a micrometre) and diffraction-limited (10 s picometre emittance) electron beam with high repetition rate (1 kHz or higher). Shorter pulses seeded by an external laser allow for more precise synchronization and controlled timing for pump–probe experiments. Compared with conventional high-gain harmonic generation (HGHG) (Yu, 1991 ▸; Yu & Ben-Zvi, 1997 ▸; Yu *et al.*, 2000 ▸; Yu & Shaftan, 2019 ▸), the echo-enabled harmonic generation (EEHG) scheme is significantly less sensitive to the typically large energy spread of a storage ring (SR). Thus, EEHG enables the possibility of SLS-based free-electron lasers (FELs) to produce intense CR pulses with durations (Stupakov, 2009 ▸; Xiang & Stupakov, 2009 ▸; Molo *et al.*, 2011 ▸; Evain *et al.*, 2012 ▸; Khan *et al.*, 2013 ▸; Khan, 2017 ▸; Feng & Zhao, 2017 ▸; Feng *et al.*, 2019 ▸; Willmott, 2019 ▸) well below the typical bunch length of tens of picoseconds. The prebunching produced by EEHG is quite different from that produced by HGHG. Compared with other schemes for implementing seeded FELs in storage rings, *e.g.* angular-dispersion-enhanced prebunching (Feng & Zhao, 2017 ▸; Feng *et al.*, 2019 ▸), the EEHG approach holds great promise not only for its compatibility with storage rings (no need for any lattice change) but also for its accessibility to higher harmonics. This is because, for the EEHG approach (Stupakov, 2009 ▸; Xiang & Stupakov, 2009 ▸), maximal bunching (*b*_*n*_) can be achieved with an energy modulation much smaller than *n*σ_E_ as required for the HGHG case, where σ_E_ is the energy spread. The EEHG configuration allows a reduced energy modulation in the second stage at the expense of a larger momentum compaction in the first stage. From harmonic 50 to harmonic 200, only about a 30–40% decrease in the achievable bunching factor is predicted while using reasonable laser power for seeding. Thanks to our recent finding (Yang *et al.*, 2022*a* ▸,*b* ▸, 2023 ▸) of allowing for different separations between the two stages, this could effectively mitigate energy-modulation-induced beam heating in σ_E_ to less than 40%, thus enabling the pump–probe capability as well as opening the door for cascaded EEHG in a novel context, toward a significant prebunching at harmonics far beyond 200. The resulting fully coherent ultra-fast photon pulses up to the tender and hard X-ray wavelengths could offer unique opportunities to conduct high-resolution phase-contrast spectroscopy on organic materials that are important in medicine, biology and bio-renewable energy materials (Mille *et al.*, 2022 ▸). Extending the pump–probe approach to hard X-rays could allow detailed studies of excited-state dynamics in organic molecules or biomolecular structures on a nanosecond to femtosecond timescale.

Based on our recent development of utilizing two straight sections (SSs) of an SR to seed coherent emission in the EUV to soft X-ray range (Yang *et al.*, 2022*a* ▸,*b* ▸), we apply the same methodology to evaluate the performance of a cascaded EEHG. This configuration not only allows one to overcome the small momentum compaction associated with the fourth-generation diffraction-limited (DL) SLSs but also provides an adequate flexibility to open the door for cascaded EEHG, which could extend the short wavelength limit to hard X-rays. Firstly, the required energy modulation can be greatly reduced, allowing CR with a much higher harmonic. Furthermore, the possibility of varying the separation between those two stages could enable a novel scheme of twin-pulse seeding in SLSs, where either the same electron bunch or two electron bunches can produce two or more distinct radiation pulses enabling the cascaded EEHG capability. To cover most of the future DL-SLSs with circumferences from a few hundred metres to several kilometres, we designed a compact cascaded EEHG beamline with the stage-1 modulator in common (*e.g.* the NSLS-II upgrade and DL-SLS in PEP tunnel). This FEL-based cascaded EEHG offers improved longitudinal coherence, output stability and time-resolved capability, and extends the spectral range to tender and hard X-ray wavelengths; thus, it can potentially broaden the scientific horizon via studying excited-state dynamics in organic molecules, allowing a greater understanding of the excited-state behavior of complex organic molecules (Stiel *et al.*, 2021 ▸; Segatta *et al.*, 2020 ▸). Furthermore, taking into account a hypothetical example of a ring with much longer straight sections, we make a side-by-side comparison among two standard SR-based FEL options: prebunching and self-amplified spontaneous emission (SASE).

## 2. Results

### 2.1. Cascaded EEHG beamline design for fourth-generation SLSs

#### 2.1.1. Design method

To take two SSs of an SR for seeding the coherent X-ray emission, a compact EEHG design utilizing the bending magnets (BMs) between these two straights as the first chicane has been implemented (Yang *et al.*, 2022*a* ▸,*b* ▸; Penn & Reinsch, 2011 ▸; Penn, 2014 ▸). A complete set of tools, *EEHG optimizer*, has been developed in our early study and successfully applied to NSLS-II and its upgrade lattices. This optimizer takes the lattice of the SR (momentum compaction α_c_ and beta function β_*x*,*y*_), the beam emittances (ɛ_*x*,*y*_), the harmonic (*h*) and the normalized energy modulation (*A*_1_ = 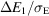 ) of stage 1 as the input parameters while all other parameters, *e.g.* the seed laser wavelengths λ_1,2_, are fixed. The output parameters are the optimized energy modulation (*A*_2_ = 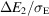 ) and momentum compaction (*R*_2_) of stage 2, which are used to generate the 6D phase space distribution. Momentum compaction of stage 1 (*R*_1_) is obtained via the equation

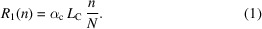

**Figure 1:**
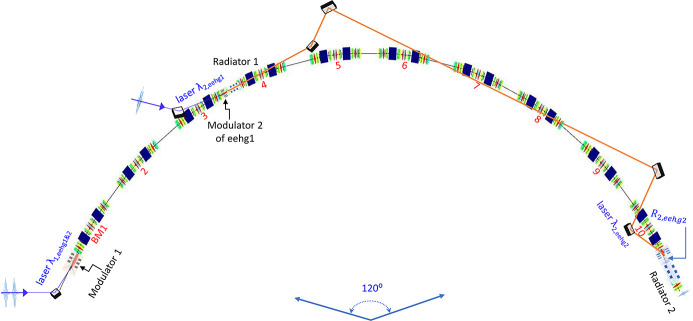
A compact design of the cascaded EEHG for the NSLS-II upgrade lattice has the stage-1 modulator in common for both beamlines. For the first cascaded EEHG beamline, named eehg1, modulator 2, chicane 2 and radiator 1 are positioned in the SS which is three BM sections downstream of the common modulator 1. An optical transport line built with four Mo/Si multilayer mirrors (PXRMS Multilayer Survey Results: https://henke.lbl.gov/cgi-bin/mldata.pl) transfers the CR pulse from radiator 1 to modulator 2 of the second cascaded EEHG beamline which is ten BM sections downstream of the common modulator 1; it is followed by chicane 2 and radiator 2 of the second beamline, named eehg2.

Here, *L*_C_, *n* and *N* are the circumference, number of BM sections between stage 1 and 2, and number of cells in the SR (Penn & Reinsch, 2011 ▸; Penn, 2014 ▸; Chao *et al.*, 2012 ▸). σ_E_ is the energy spread, which is fixed in a SR-based FEL (*e.g.* 8 × 10^−4^ for the NSLS-II upgrade lattice) by the equilibrium between radiation damping and energy diffusion (Feng *et al.*, 2019 ▸); Δ*E*_1_ and Δ*E*_2_ are the energy modulation amplitudes of stage 1 and 2, respectively. This EEHG optimizer is directly applicable to the design of the cascaded EEHG. Then, a *GENESIS* (Reiche, 1999 ▸) simulation is applied to obtain the CR properties within a usually short undulator distance, limited by the available space of a SR (Dierker, 2007 ▸; Yu *et al.*, 2022 ▸). Illustration of the compact version of the cascaded EEHG/two-stage EEHG prebunching includes the common stage-1 modulator, which is shared between the first and second cascaded EEHG beamlines, and chicane 1, modulator 2, chicane 2 and radiator 1 which only belong to the first beamline; then, the CR output from radiator 1 provides the input seed to the stage-2 modulator of the second beamline, as shown in Fig. 1 ▸.

#### 2.1.2. Critical parameters determining EEHG beamline performances

The main lattice parameters determining EEHG performances are the momentum compaction, beam emittances and beta functions (Yang *et al.*, 2022*a* ▸,*b* ▸), and they are often varied over a broad range among the SLSs. Regarding the fourth-generation SLSs, momentum compactions are often a few to ten times smaller than their third-generation counterparts. To overcome the small momentum compaction intrinsically associated with any fourth-generation DL-SLS (Yu *et al.*, 2022 ▸; Wang *et al.*, 2019 ▸; Li *et al.*, 2021 ▸; APS, 2019 ▸; Borland, 2000 ▸), separating stages 1 and 2 with a few more BM sections has been demonstrated to be an effective way to increase the momentum compaction of chicane 1 (Yang *et al.*, 2022*b* ▸, 2023 ▸). This allows further reductions of the energy modulation *A*_1_ from 2.5 (Yang *et al.*, 2022*a* ▸) to <2, not only mitigating the required seed laser powers (Yang *et al.*, 2023 ▸) but also reducing the beam heating in the energy spread below 100%. To cover hard X-rays with a photon energy up to 10 keV, one could utilize the cascaded EEHG beamline design with the stage-1 modulator in common, just in case there are space limitations, then construct the rest of the beamline, whereas the CR output from the first stage-2 modulator and radiator pair with harmonic *h*_1_ provides the input seed to the second stage-2 modulator with harmonic *h*_2_ (Feng & Zhao, 2010 ▸; Zhao *et al.*, 2016 ▸, 2017 ▸; Fan *et al.*, 2022 ▸). Hence, the second radiator could potentially generate a very high harmonic regarding the seed laser wavelength λ_1_ of the common stage-1 modulator, *e.g.* *h* = 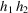 = 20(25–100) ≃ 500–2000, toward the tender and hard X-ray wavelengths. Here, we choose the seed-laser wavelength λ_1_ = 256 nm for the common stage-1 modulator. To achieve an adequate CR power for seeding the second EEHG beamline as well as to keep the harmonic *h*_2_ below 200, the harmonic *h*_1_ of the first beamline should be around 20 preferably; thus, the harmonic *h*_2_ only needs to be in the range 25–100 to achieve a final CR wavelength of 0.51–0.128 nm. Moreover, the most important reason to choose the harmonic *h*_1_ = 20 is due to consideration of available transport line optics with minimum losses. In the EUV spectrum, the reflectivity at a wavelength 12.8 nm is generally quite high (https://henke.lbl.gov/cgi-bin/mldata.pl), where Mo/Si multilayers have a reflectivity approaching ∼83% at a grazing angle of 45°. Thus, an optical transport line can be built based on four such mirrors to achieve a transmission efficiency of 48% (= 0.83^4^) (https://henke.lbl.gov/cgi-bin/mldata.pl).

To study the feasibility of the cascaded EEHG option for NSLS-II, its future upgrade and a recent proposed DL-SLS in PEP tunnel (named SDLS) (Raimondi *et al.*, 2023 ▸; Yu *et al.*, 2022 ▸; Wang *et al.*, 2019 ▸; Li *et al.*, 2021 ▸; APS, 2019 ▸; Borland, 2000 ▸), we have collected the crucial parameters determining the EEHG performance and show them in Table 1 ▸. *R*_1_ (1–10 mm) for one BM section and β (1–5 m) cover the complete parameter space. For the NSLS-II lattice, we mainly focus on the future upgrade, and fix ɛ_*x*_ = 25 pm, ɛ_*y*_ = 5 pm by assuming a 20% coupling and electron beam energy *E* = 3 GeV.

#### 2.1.3. Energy modulation optimization

There is a trade-off between energy modulation and prebunching, as maximizing prebunching often requires increased energy modulation, which leads to a higher final energy spread for the part of the beam that interacts with the external laser, reducing peak power. The energy modulation needs to be increased substantially with the increase of the harmonic, especially for the fourth-generation SLSs with small momentum compactions (Yang *et al.*, 2022*a* ▸,*b* ▸). In a short radiator, which is the case for most SR-based FELs, the final CR power is negatively correlated to the final energy modulation of the beam slice. Thus, we focus on a specific case where the initial prebunching provides the main portion of the total radiation but having negligible exponential gain. To overcome the small momentum compaction intrinsically associated with any fourth-generation SLS, we will utilize our recent finding that, by separating stage 1 and 2 with a few extra BM sections, the momentum compaction of stage 1 can be significantly increased as well as broadly tunable. The momentum compaction of stage 1 (*R*_1_) is linearly proportional to the number of BM sections (*n*) between those two stages (Yang *et al.*, 2023 ▸; Chao *et al.*, 2012 ▸),

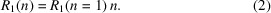

Here, *R*_1_(*n* = 1) is the momentum compaction of one BM section, which can be estimated via equation (1) with *n* = 1. As a result, the energy modulation required by any fourth-generation SLS can be greatly reduced, especially for high harmonics.

Particle tracking simulations that consider second-order transport effects with quantum excitation and radiation damping being turned on were used to confirm that with an increased number of BM sections, up to ten, for the APS-U lattice there exists negligible degradation of the modulated longitudinal phase space (LPS) (Yang *et al.*, 2023 ▸). To study in detail how well the longitudinal phase space distribution after the stage-1 energy modulation is preserved after passing through an increased number of BM sections, we performed particle-tracking studies (Terebilo, 2001 ▸) based on the APS-U lattice (APS, 2019 ▸; Borland, 2000 ▸) with *A*_1_ = 2.5. Quantum excitation and radiation damping are turned on in the particle tracking setup. First, we only track a beam slice with a longitudinal size of one seed laser wavelength (λ_1_ = 250 nm). The longitudinal phase space distribution right after the stage-1 energy modulation is used as the initial distribution; then, an equilibrium transverse distribution is added to this longitudinal phase space distribution with a random mixing of the transverse and longitudinal particle index. The simulation confirms that the LPS with the stage-1 energy modulation is perfectly preserved after passing through an increased number of BM sections up to ten, since the ratio of the root mean square (RMS) bunch length and the corresponding energy spread is linearly proportional to *n*, with the slope as the momentum compaction of one BM section, and the normalized energy spread stays constant. Also, we track the energy-modulated electron beam slice via stage 1 with a width up to 500λ_1_. So, we can have a LPS distribution that is the closest replica of reality. We compare the LPS evolving through various BM sections via a particle tracking simulation with the calculated distribution via analytical formula (Stupakov, 2009 ▸; Xiang & Stupakov, 2009 ▸), and find that they are identical (Yang *et al.*, 2023 ▸).

Moreover, CR with a hard X-ray wavelength is more sensitive to energy-spread-induced de-bunching, which is proportional to the product of the total energy spread after the modulation and the dispersion of the radiator. This can be partially ameliorated by aiming for optimal bunching to occur in the center of the radiator. Whenever the initial prebunching is amplified the most by the CR process, maximal bunching should be achieved somewhat before but very close to the midpoint (Yang *et al.*, 2022*a* ▸). The purpose of the design optimization becomes the search for an optimal energy modulation regarding a specific harmonic, which provides the maximum growth of the CR power as well as simultaneously mitigating the rapid de-bunching induced by the large energy spread. The energy-spread-induced pathlength difference ( 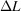 ) in an undulator can be approximately estimated via equation (3),

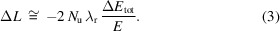

It is valid only in the absence of gain, which is appropriate in this case. To largely preserve the initial prebunching generated by EEHG, 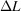 needs to be kept significantly smaller than the radiation wavelength λ_r_ (*e.g.* 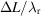 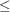 0.3) for as long as possible in the radiator. Here, 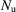 = 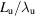 refers to the number of undulator periods in the radiation stage, and *L*_u_ and λ_u_ are the undulator length and period, respectively.

For an SR-based EEHG FEL, the undulator length is often limited to a few metres due to the available space of a SR. Regarding the short wavelength limit toward hard X-rays, the minimum initial energy modulation can be achieved at the cost of less prebunching; thus, slower CR power growth as well as slower de-bunching evolves along the undulator distance; eventually, the CR output could reach a significantly higher peak power with optimal prebunching. This is the reason why the optimal energy modulation of stage-1, *A*_1_, is preferred to be 1.3 (see details in the next few paragraphs) (Yang *et al.*, 2022*a* ▸,*b* ▸). Also, *A*_1_ would be limited by the LPS diffusing, which causes a smearing out of those energy stripes formed after chicane 1 of stage 1 (see Section 2.1.4 for details).

**Figure 2:**
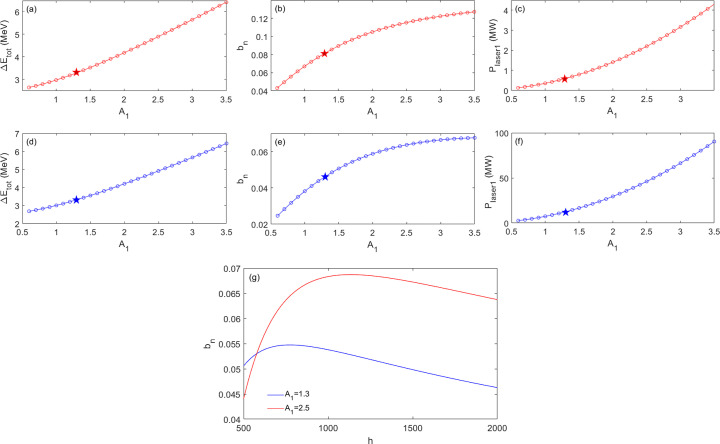
For the NSLS-II upgrade lattice with harmonic 2000 and electron beam energy 3 GeV, regarding the first part of the cascaded EEHG, (*a*) total energy modulation Δ*E*_tot_, (*b*) prebunching *b*_*n*_ and (*c*) required stage-1 seed laser power as a function of *A*_1_. The required stage-2 seed laser power, normalized energy modulation *A*_2_ and momentum compaction of chicane 2 stay nearly constant, 0.162 MW, 0.254 and −212 µm, respectively, despite the variation of *A*_1_. Similarly, for the second part of the cascaded EEHG, (*d*) total energy modulation Δ*E*_tot_, (*e*) prebunching *b*_*n*_ and (*f*) required stage-1 seed laser power as a function of *A*_1_. The required stage-2 seed laser power, normalized energy modulation *A*_2_ and momentum compaction of chicane 2 stay nearly constant, 6.9 MW, 0.376, and −7.02 µm, respectively. (*g*) Prebunching *b*_*n*_ as a function of the cascaded EEHG harmonic *h* for two different values of *A*_1_: 1.3 (blue) and 2.5 (red).

As a result of the optimization process regarding a specific harmonic 2000 for the first part of the cascaded EEHG, *R*_1_ is fixed to 4.2 mm (three BM sections of the NSLS-II upgrade lattice). Figures 2 ▸(*a*) and 2 ▸(*b*) show the total energy modulation 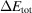 and the prebunching *b*_*n*_ as a function of the stage-1 modulation amplitude (*A*_1_), respectively. 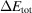 increases from 2.6 to 6.4 MeV with *A*_1_ increasing from 0.6 to 3.5; *b*_*n*_ almost reaches its saturation with a value of 12.0% when *A*_1_ ≥ 2.5. Figure 2 ▸(*c*) shows the required stage-1 seed laser power, which can be provided by a commercially available laser system with a wavelength of 256 nm (Del Mar Photonics, https://www.dmphotonics.com/index.htm; MKS Instruments Light & Motion Division: Spectra-Physics, https://www.spectra-physics.com). Despite the variation of *A*_1_, the required stage-2 seed laser power, normalized energy modulation *A*_2_ and momentum compaction of chicane 2 stay almost constant: 0.162 MW, 0.254 and −212 µm, respectively. Red stars in Figs. 2 ▸(*a*)–2(*c*) highlight the optimized results for *A*_1_, 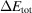 , *b*_*n*_ and *P*_laser1_ of the first beamline.

Similarly, for the second part of the beamline with a fixed *R*_1_ = 14.0 mm (ten BM sections) for the reason explained in Section 2.1.4, Figs. 2 ▸(*d*), 2 ▸(*e*) and 2 ▸(*f*) show the total energy modulation 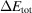 , prebunching *b*_*n*_ and required stage-1 seed laser power as a function of *A*_1_, respectively. 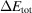 increases from 2.6 to 6.4 MeV with *A*_1_ increasing from 0.6 to 3.5; *b*_*n*_ almost reaches its saturation with a value of 6.2% when *A*_1_ ≥ 2.5. Similarly, *A*_1_ should stay below 2.5; the CR output reaches its maximum when *A*_1_ = 1.3 since for such a high harmonic the CR process is dominated by energy-spread-induced rapid de-bunching which prefers a small energy modulation. The required stage-2 seed laser power, normalized energy modulation *A*_2_ and momentum compaction of chicane 2 are 6.9 MW, 0.376 and −7.02 µm, respectively. Blues stars in Figs. 2 ▸(*d*)–2(*f*) highlight the optimized parameters for *A*_1_, 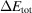 , *b*_*n*_ and *P*_laser1_ of the second beamline.

It is evident that the required stage-2 seed laser power for the cascaded EEHG beamline stays almost constant (∼6.9 MW) despite different values of *A*_1_, mainly due to the similarity of the stage-2 modulation with HGHG (*A*_2_ and *R*_2_ are adjustable together for a specific harmonic). The harmonic *h*_1_ of radiator 1 is chosen to be 20 for the purpose of mitigating the energy loss (∼50%) during the transferring of the CR pulse from radiator 1 to the second stage-2 modulator. The output of radiator 1 needs to be at least two times the required stage-2 seed laser power for the second part of the beamline, 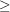 2 × 6.9 MW. A *GENESIS* simulation performed in our earlier study (Yang *et al.*, 2022*a* ▸,*b* ▸) shows that a peak power of 23.5 MW can be achieved with a radiator length of 3.5 m at harmonic *h*_1_ = 20. Since the CR power scales quadratically with the radiator length *L*_u_ (Yang *et al.*, 2022*a* ▸,*b* ▸) by a factor 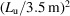 , radiator 1 only needs to be 2.7 m long to generate the required CR power [ 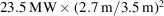 ≃ 14 MW] for seeding the second stage-2 modulator. We assume that the stage-1 energy modulations for the first and second cascaded EEHG beamlines are the same, *A*_1,eehg1_ = *A*_1,eehg2_ = *A*_1_. Figure 2 ▸(*g*) shows the prebunching *b*_*n*_ as a function of the harmonic of the cascaded EEHG (*h*) with two different values of the stage-1 modulation amplitude, *A*_1_ = 1.3 (blue) and *A*_1_ = 2.5 (red). It implies that a 30% gain in the prebunching for harmonic >1000 requires a significant increase (≥50%) of the final energy spread. Moreover, the bunching factor only decreases mildly (10–20%) with an increase of the harmonic from 1000 to 2000, thus EEHG can access very high harmonics.

#### 2.1.4. Important factors affecting EEHG performance

A fundamental aspect of the EEHG process is that the microbunching from the laser modulation is quickly suppressed after it passes through the first chicane with a large momentum compaction *R*_1_, as the energy modulation folds over on itself multiple times. Only in the second stage does such an impact resurface (named ‘echo’). We considered the effects of incoherent and coherent synchrotron radiation (ISR and CSR), similar to Xiang & Stupakov (2009 ▸). The ISR-induced energy spread for ten BM sections of the NSLS-II upgrade lattice with *R*_1_ = 14 mm is about 10 keV (Yang *et al.*, 2023 ▸; Yu *et al.*, 2022 ▸). Compared with the separation between adjacent energy bands estimated by equation (4) (Yang *et al.*, 2023 ▸),

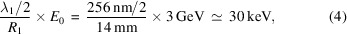

one could expect a minor degradation of the bunching factor. In the first chicane, since the beam is not micro-bunched, the CSR effect should be negligible; in the second chicane, the beam is briefly micro-bunched only at the last dipole (often 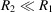 ). The situation is quite similar to that considered by Xiang & Stupakov (2009 ▸). Moreover, since the peak current of a SR is significantly lower [*e.g.* <300 A in the NSLS-II upgrade compared with 800 A in a linac (Xiang & Stupakov, 2009 ▸)], the CSR effect should be much weaker.

In the cascaded EEHG process considered here, the electron bunch is modulated by a seed laser with a wavelength of 256 nm. The energy modulation will turn into a density modulation for a brief period in the first BM section, but the density modulation then rapidly folds over on itself multiple times along the *z*-coordinate and effectively smooths the density modulation, leaving only internal structure hidden in the longitudinal phase space. Thus, the CSR effect on the beam should be minor. Instead, the ISR effect could have a much more significant impact on the cascaded EEHG process, especially for the second beamline since the separation between adjacent energy bands is linearly proportional to the seed laser wavelength λ_1_ and inversely proportional to the momentum compaction *R*_1_ of stage 1 [equation (4)]. The energy-band separation could be 20 times smaller if one chooses 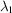 = 256 nm/20 = 12.8 nm compared with the case with λ_1_ = 256 nm; hence, the ISR effect could potentially wash out those energy stripes. To mitigate the ISR effect, one would prefer λ_1_ = 256 nm for both beamlines, which leads to a similar design of the compact cascaded EEHG shown in Fig. 1 ▸.

Moreover, regarding a particular design of the EEHG especially for high harmonics, it is a trade off between maximizing the CR output via minimizing the energy modulation and mitigating the ISR effect via maximizing the energy stripe separation. The final energy spread can approximately be given by equation (5) (Yang *et al.*, 2023 ▸),

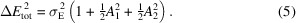

**Figure 3:**
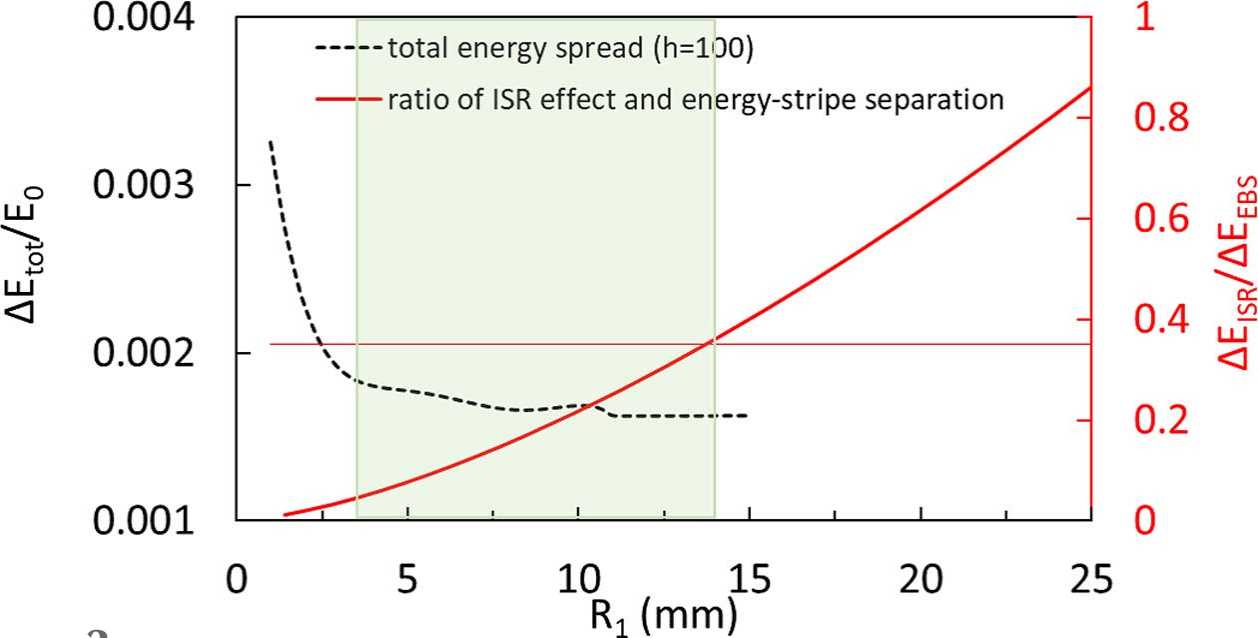
The normalized final energy spread as a function of the momentum compaction of stage 1 is shown for harmonic 100 as the primary *y*-axis (black) (Yang *et al.*, 2022*b* ▸). The ratio of ISR-induced energy spread increase ( 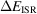 ) over the energy band separation ( 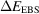 ) is plotted as the secondary *y*-axis (red). We assume the relevant electron beam, seed laser and modulator parameters in simulations based on the NSLS-II upgrade lattice: the electron beam with energy 3 GeV, emittances ɛ_*x*_ = 25 pm and ɛ_*y*_ = 5 pm and relative energy spread 8 × 10^−4^; the seed laser wavelength λ_1_ = 256 nm, and peak power depending on harmonic numbers below 10 GW; the modulator of stage 1 with a period of 20 cm and number of periods 10.

Minimizing the energy modulation would result in a higher CR output power (Yang *et al.*, 2022*b* ▸), and this often requires a large *R*_1_ (primary *y*-axis as the black curve in Fig. 3 ▸); instead, to mitigate the ISR effect, maximizing the energy-band separation prefers a small *R*_1_ [equation (4)]. Here, with a fixed λ_1_ = 256 nm, the optimization becomes searching for a particular parameter set (*A*_1_, *R*_1_, *A*_2_) to maximize the CR output power as well as its coherences.

As shown in Fig. 3 ▸, the optimal choice of *R*_1_ needs to be in the range 3–14 mm (green highlight area) for the NSLS-II upgrade lattice due to the following considerations: (i) the energy modulation needs to be comparable with or below the initial energy spread; and (ii) the ISR effect needs to be minor by keeping the ratio of the ISR-induced energy spread increase ( 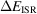 ) and the energy-band separation ( 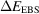 ) below 0.35 (red horizontal line in Fig. 3 ▸). This explains why for a compact design of the cascaded EEHG in the NSLS-II upgrade case we choose the two-stage separations for the first and second beamlines to be three and ten BM sections, which correspond to *R*_1_ = 4.2 mm and 14 mm, respectively.

#### 2.1.5. Cascaded EEHG beamline output peak power

The optimized cascaded EEHG parameters for the first and second beamlines are summarized in Table 2 ▸. For the second part of the cascaded EEHG with a photon energy of 10 keV, the optimized results are *A*_1_ = 1.3, *A*_2_ = 0.376, *R*_1_ = 14.0 mm, *R*_2_ = −7.02 µm and 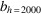 = 0.046.

**Figure 4:**
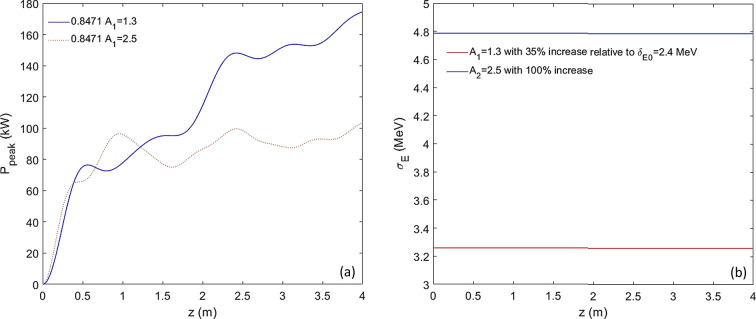
Regarding the cascaded EEHG with harmonic 2000, average β_*x*,*y*_ = 1.7 m and *I*_peak_ = 300 A, (*a*) radiator-2 is set to lase in the second harmonic with an undulator period of 10 mm and optimal detuning *K*_u,helical_ = 0.8471; the peak power as a function of the undulator distance is shown for two cases, *A*_1_ = 1.3 (blue) and *A*_1_ = 2.5 (red). (*b*) RMS energy spread as a function of the undulator distance is shown for two cases, *A*_1_ = 1.3 (red) and 2.5 (blue). Regarding the peak current *I*, the final CR power can be scaled quadratically by the factor 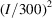 (Yang *et al.*, 2022*a* ▸,*b* ▸).

To achieve the value of *A*_2_, the first part of the cascaded EEHG must produce 14 MW at a wavelength of 12.8 nm, which requires only 2.7 m of radiating undulator. The number of BM sections (*n*) between the stage-1 modulator and the second stage-2 modulator is determined by the optimal *R*_1_ = 14.0 mm. The reason why such an *R*_1_ (= 14.0 mm) is chosen is because the number of energy stripes formed in the LPS after the stage-1 modulation is linearly proportional to the product *A*_1_ *R*_1_, which is mainly determined by the harmonic. Thus, a small *A*_1_ sets the constraint of *R*_1_ being large. However, the maximum allowed *R*_1_ is restricted by the ISR effect (Fig. 3 ▸), which sets the upper limit *R*_1_ ≤ 14 mm. A helical undulator is often used to maximize the CR output power. However, a helical undulator at the fundamental will need a 5 mm undulator period and be challenging to engineer, so instead we consider an undulator period of 10 mm for the final radiator, named radiator 2, which can be set to lase at the second harmonic. As a result, the CR peak power as a function of the undulator distance is significantly reduced, as shown in Fig. 4 ▸(*a*). The maximum peak power that can be achieved in an undulator distance of 4 m is only 170 kW.

We numerically confirm via *GENESIS* simulations that for the cascaded EEHG regarding extremely high harmonics the energy-spread-induced rapid de-bunching prefers a much smaller initial energy modulation 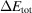 = 3.3 MeV (*A*_1_ = 1.3) with 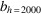 ≃ 4%, compared with 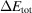 = 5.0 MeV (*A*_1_ = 2.5) with 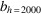 = 6%. Thanks to the CR process, the electron beam energy heating effect along the undulator can be negligible, as shown in Fig. 4 ▸(*b*). This configuration does rely on a peak current of 300 A, which is significantly above the design value, but in the tender X-ray regime such a large current would not be required.

#### 2.1.6. Timing requirements

We are currently considering applying twin-pulse seeding to the same electron bunch for the cascaded EEHG design. Thus, one must match the time-of-flight of the electron bunch from radiator 1 to the second modulator 2 (*e.g.* *n*_2_ − *n*_1_ = 10 − 3 = 7 periods of the NSLS-II upgrade lattice) with the time for the seed laser pulse traveling from radiator 1 to the second modulator 2 with a precision on the level of a small fraction (<10% 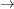 <0.1 ps) of the modulated portion (*e.g.* 1 ps) of the electron bunch. Here, *n*_1_ and *n*_2_ are the number of BM sections between stage-1 and stage-2 modulators regarding the first and second cascaded EEHG beamlines, respectively. If there is any obstacle in designing such an optical delay line to match the seed laser pulse with the time-of-flight of the electron bunch, often a few µs, one can always choose two different electron bunches with an adjustable delay in the range of an RF period (*e.g.* 2 ns for the NSLS-II upgrade) up to a revolution period of a SR (2.6 µs). So far, we have not foreseen any showstopper for making such a matching between the seed laser pulse and the electron bunch in the second modulator 2 of the cascaded EEHG. The optical delay can be built in-vacuum to avoid air turbulence achieving the required µrad spatial pointing jitter, which is limited by the transverse walk-off between the seed laser and the electron bunch not exceeding a small fraction of the beam size.

## 3. Design FEL beamline for a future upgrade large-size SR

### 3.1. Case 1: coherent seeding via cascaded EEHG

**Figure 5:**
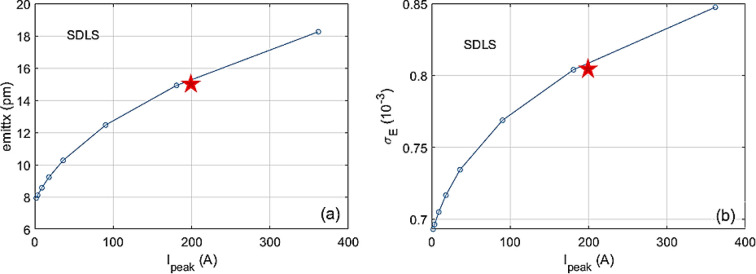
For the SDLS lattice, the IBS effect has been considered: emittance (*a*) and normalized energy spread (*b*) as a function of electron beam peak current.

Future large-scale SRs with long straight sections, *e.g.* PETRA IV (Schroer *et al.*, 2019 ▸) or a DL-SLS in the PEP tunnel (Raimondi *et al.*, 2023 ▸), could take advantage of the full potential of the cascaded EEHG scheme. In this section we examine the FEL performance with parameters of the SDLS, a recent storage-ring lattice design for the PEP tunnel (Raimondi *et al.*, 2023 ▸). The relevant parameters determining the EEHG performances are listed in Table 1 ▸. The intra-beam scattering (IBS) effect is taken into account (Raimondi *et al.*, 2023 ▸); emittance and energy spread of the electron beam increase significantly with the increase of the peak current, as shown in Figs. 5 ▸(*a*) and 5 ▸(*b*), respectively. Thus, the horizontal and vertical emittances ɛ_*x*,*y*_ are equal, 15 pm, and the energy spread is 8 × 10^−4^ at the peak current *I*_peak_ = 200 A and beam energy *E* = 5 GeV.

It is evident that the cascaded EEHG option could benefit SDLS much more than the NSLS-II upgrade for the following main reasons: (i) higher electron beam energy of 5 GeV; (ii) much longer SS of length 122 m; (iii) lower emittances in both *x* and *y* directions of 15 pm; and (iv) larger momentum compaction for each BM section of 9.6 mm. In particular, a 122 m-long SS could allow the cascaded EEHG beamline to be built ideally with two independent stage-1 modulators, stage-2 modulators and radiators. However, ISR is the most significant effect that could potentially smear out these energy stripes. To mitigate this potentially severe impact, one must keep the ratio of the ISR-induced energy spread increase ( 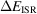 ) and the energy-band separation ( 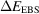 ) below 0.35. Since 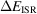 per BM section is calculated to be 19.8 keV, similar to the method used by Xiang & Stupakov (2009 ▸) based on an average bending radius of 93 m in the SDLS lattice (Raimondi *et al.*, 2023 ▸), the expression 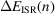 ≃ *n* ^1/2^ × 19.8 keV can be applied to estimate 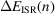 when *n* = 1, 2,…, 6, which is the total number of cells in the SDLS; the energy-stripe separation can be calculated via equation (4),

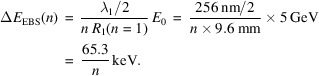

**Figure 6:**
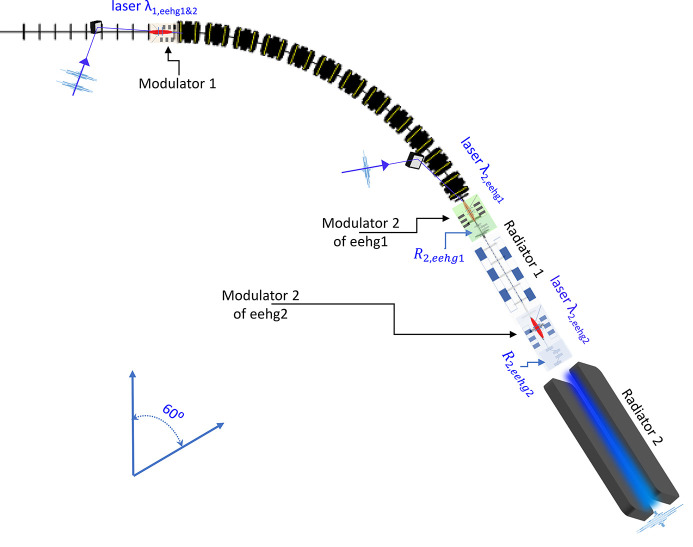
Layout of the cascaded EEHG beamline design for the SDLS lattice: the common sections include modulator 1 and chicane 1 for the two cascaded EEHG beamlines, named eehg1 and eehg2. A BM section with bending angle of 60° becomes chicane 1 for both eehg1 and eehg2 beamlines. It is followed by modulator 2, chicane 2 and radiator 1 of eehg1. The output of radiator 1 becomes the seed laser 2 of eehg2, which interacts with the electron beam in modulator 2; afterwards, chicane 2 turns those energy stripes into current spikes, which generate the CR output in radiator 2 of eehg2.

As a result, the criterion 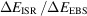 ≃ 0.3*n* ^1.5^ 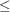 0.35 can only be satisfied when *n* = 1. Thus, one must apply a similar design to the NSLS-II upgrade case. With the modulator 1 in common for both beamlines, each modulator 2 and radiator pair are unique for each beamline and these two pairs are placed in the same SS, which is one BM section downstream of the common modulator 1, as shown in Fig. 6 ▸. This arrangement can potentially eliminate the optical transport line between radiator 1 and the second modulator 2, greatly reducing the required stage-2 seed laser power of the second beamline, which is provided by the output of radiator 1.

**Figure 7:**
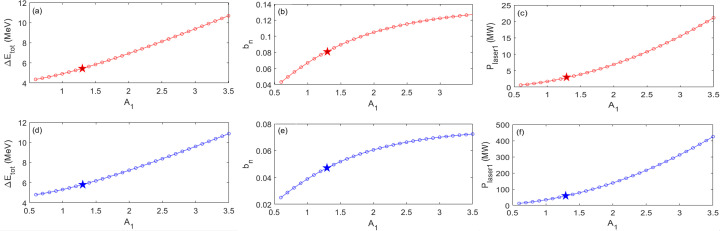
For the SDLS lattice (Raimondi *et al.*, 2023 ▸) with harmonic 2000 and electron beam energy 5 GeV, regarding the first cascaded EEHG beamline, (*a*) total energy modulation Δ*E*_tot_, (*b*) prebunching *b*_*n*_ and (*c*) required seed laser power of stage 1 as a function of *A*_1_. The required seed laser power of stage 2, normalized energy modulation *A*_2_ and momentum compaction of chicane 2 stay nearly constant at 0.153 MW, 0.149 and −483 µm, respectively, despite the variation of *A*_1_. Similarly, for the second cascaded EEHG beamline, (*d*) total energy modulation *ΔE*_tot_, (*e*) prebunching *b*_*n*_ and (*f*) required seed laser power of stage 1 as a function of *A*_1_. The required seed laser power of stage 2, normalized energy modulation *A*_2_ and momentum compaction of chicane 2 are 71.5 MW, 0.548 and −4.83 µm, respectively. Based on the quadratically scaling relationship (Yang *et al.*, 2022*a* ▸,*b* ▸), radiator 1 needs to be 6.2 m long in order to produce the required CR power [ 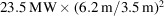 ≃ 74 MW] for seeding.

As a result of optimization regarding a specific harmonic 2000, for the first part of the cascaded EEHG beamline with *R*_1_ = 9.6 mm (one BM section), Figs. 7 ▸(*a*), 7 ▸(*b*) and 7(*c*) show the total energy modulation 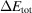 , prebunching *b*_*n*_ and required stage-1 seed laser power as a function of *A*_1_, respectively. 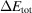 increases from 4.4 to 10.7 MeV with *A*_1_ increasing from 0.6 to 3.5; *b*_*n*_ almost reaches saturation with a value of 12.0% when *A*_1_ is equal to 2.5. Thus, *A*_1_ should stay below 2.5 with an optimal value of 1.3, corresponding to 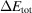 = 5.4 MeV. Red stars in Figs. 7 ▸(*a*) to 7(*c*) highlight the optimized settings for *A*_1_, 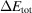 , *b*_*n*_ and *P*_laser1_ of the first beamline.

For the second part of the cascaded EEHG beamline with *R*_1_ = 9.6 mm (one BM section), Figs. 7 ▸(*d*), 7 ▸(*e*) and 7(*f*) show the total energy modulation 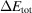 , prebunching *b*_*n*_ and required stage-1 seed laser power as a function of *A*_1_, respectively. 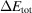 increases from 4.8 to 10.9 MeV with *A*_1_ increasing from 0.6 to 3.5; *b*_*n*_ almost reaches saturation with a value of 6.6% when *A*_1_ is equal to 2.5. Similarly, *A*_1_ should stay below 2.5 with an optimal value of 1.3, corresponding to 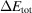 = 5.7 MeV. Blues stars in Figs. 7 ▸(*d*) to 7(*f*) highlight the optimized results for *A*_1_, 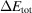 , *b*_*n*_ and *P*_laser1_ of the second beamline.

For the second cascaded EEHG beamline with a photon energy of 10 keV, the optimized parameters are *A*_1_ = 1.3, *A*_2_ = 0.548, *R*_1_ = 9.6 mm, *R*_2_ = −4.83 µm and 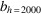 = 0.047. The number of BM sections (*n*) between the stage-1 and stage-2 modulators is determined by the fixed *R*_1_ = 9.6 mm (one BM section of the SDLS lattice). To achieve the desired modulation, *A*_2_, the required seed laser power at 12.8 nm from the first part of the cascaded EEHG is 71.5 MW. Based on the quadratically scaling relationship, radiator 1 needs to be 6.2 m long to produce the required CR power [ 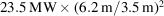 ≃ 74 MW] for seeding. For radiator 2 to produce 10 keV radiation, if the radiator is set to lase at the fundamental mode, the undulator parameters (period and *K*-factor) need to be λ_u_ = 10 mm and *K*_u,helical_ = 1.1770. The optimized cascaded EEHG parameters for the first and second beamlines are summarized in Table 3 ▸.

**Figure 8:**
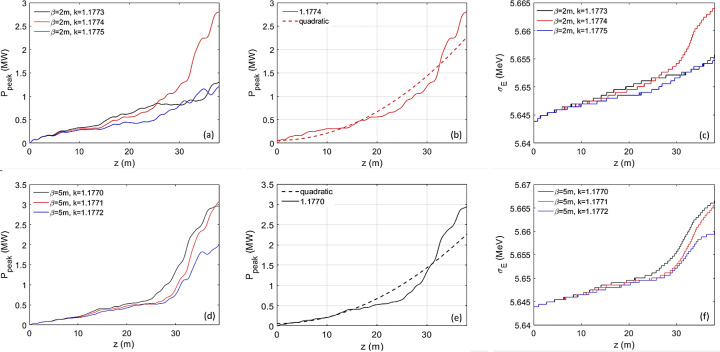
Regarding the cascaded EEHG with harmonic 2000 and *I*_peak_ = 200 A, the radiator-2 is set to lase in the fundamental with an undulator period of 10 mm when the averaged β_*x*,*y*_ = 2 m, (*a*) the peak power as a function of the undulator distance is shown for the optimal detuning *K*_u,helical_ = 1.1774 (red), unoptimized detuning-1 *K*_u,helical_ = 1.1773 (black) and detuning-2 *K*_u,helical_ = 1.775  (blue). (*b*) The peak power as a function of the undulator distance for the optimal detuning *K*_u,helical_ = 1.1774 can be fitted quadratically with the undulator distance up to 38 m. (*c*) RMS energy spread as a function of the undulator distance is shown for those three cases, *K*_u,helical_ = 1.1774 (red), 1.1773 (black) and 1.775 (blue). Similarly, for β_*x*,*y*_ = 5 m, (*d*) the peak power as a function of the undulator distance is shown for optimal detuning *K*_u,helical_ = 1.1770 (black) and *K*_u,helical_ = 1.1771 (red) and unoptimized detuning *K*_u,helical_ = 1.772 (blue). (*e*) The peak power as a function of the undulator distance for the optimal detuning *K*_u,helical_ = 1.1770 can be fitted quadratically with the undulator distance up to 38 m. (*f*) RMS energy spread as a function of the undulator distance is shown for the three cases *K*_u,helical_ = 1.1770 (black), 1.1771 (red) and 1.772 (blue).

A helical undulator is used to maximize the CR output power. First, we assume β_*x*_ = β_*y*_ = 2 m. The CR output peak power as a function of the undulator distance is shown in Fig. 8 ▸(*a*) with optimal detuning of 1.1774 (red), unoptimized detuning-1 of 1.1773 (black) and unoptimized detuning-2 of 1.1775 (blue). There exists an optimal detuning where the CR power is maximized [red curve in Fig. 8 ▸(*a*)]. The undulator with period of 10 mm is currently available. Similar simulations are performed when β_*x*_ = β_*y*_ = 5 m, and the results are shown in Figs. 8 ▸(*d*), 8 ▸(*e*) and 8(*f*), respectively. We numerically confirm via *GENESIS* simulations that, for the cascaded EEHG regarding an extremely high harmonic, the energy-spread-induced rapid de-bunching prefers an optimized small initial energy modulation 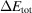 = 5.7 MeV (*A*_1_ = 1.3) with 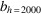 = 4.7%.

The CR peak power can reach 3 MW at a photon energy of 10 keV with undulator distances of 38 m in both cases, β_*x*,*y*_ = 2 m and β_*x*,*y*_ = 5 m; also, the CR power increases with the undulator distance close to a quadratic relationship, as shown in Figs. 8 ▸(*b*) and 8 ▸(*e*). This quadratic feature can be understood by the following: (i) we can write a function describing the power growth in the FEL amplifier, starting from a prebunched beam with bunching factor *b*_*n*_; and (ii) the requirement is that the function has the correct quadratic growth in the first part of the undulator and grows exponentially after the undulator distance exceeds the threshold 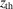 = 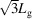 > 60 m (Xie, 1995 ▸; Giannessi, 2015 ▸). The 3D gain length 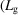 ≃ 60 m) is estimated via Xie’s fitted formula (Xie, 1995 ▸). Thus, the gain curve can be fit quadratically with an undulator distance of about 40 m [Figs. 8 ▸(*b*) and 8 ▸(*e*)] via equation (6) (Giannessi, 2015 ▸),

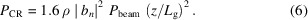

The calculated coefficient agrees reasonably well with the fitted value (discrepancy 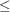 20%). Here, ρ and *P*_beam_ are the FEL Pierce parameter and the peak power of the beam, respectively. In addition to the 40% increase of the initial energy spread due to the energy modulation, the CR process only brings <0.5% beam heating within the undulator distance of 38 m [Figs. 8 ▸(*c*) and 8 ▸(*f*)]. Also, the simulation result is consistent with the feature that the gain length stays nearly constant when the beta functions are varied in the range from 2 to 5 m (see Section 3.2 for details).

**Figure 9:**
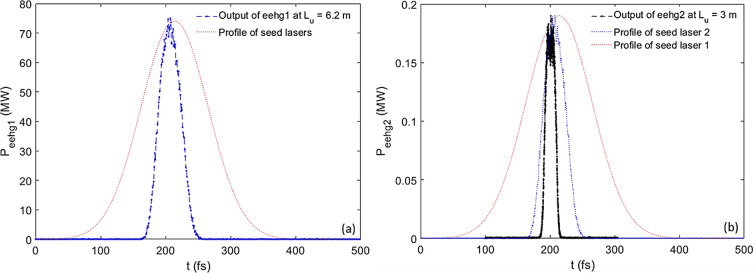
(*a*) Regarding the eehg1 beamline, intensity profiles of seed lasers 1 and 2 are the same (red curve). The CR output is shown as the blue curve. (*b*) For eehg2, the intensity profiles of seed lasers 1 and 2 and the CR output are shown as red, blue and black curves, respectively.

We also performed time-dependent *GENESIS* simulations (Reiche, 1999 ▸) based on the parameters of seed lasers, modulators and radiators listed in Table 3 ▸. To guarantee the full coherence, we limit the radiator-2 length to a few metres, *e.g.* *L*_u_ = 3 m. The simulation window covers both seeded and unseeded regions. Regarding eehg1, pulse durations of seed lasers 1 and 2 are the same, σ_t_ = 51.6 fs, shown as the red curve in Fig. 9 ▸(*a*). The CR output from radiator 1 [blue curve in Fig. 9 ▸(*a*)] has a wavelength of 12.8 nm, peak power of 74 MW and pulse duration of 23 fs; hence, it will be applied as the input seed to the stage-2 modulator of eehg2, shown as the blue curve in Fig. 9 ▸(*b*). We assume a similar pulse duration of seed laser 1 [red curves in Figs. 9 ▸(*a*) and 9 ▸(*b*)] for both eehg1 and eehg2. The CR output from radiator 2 [black curve in Fig. 9 ▸(*b*)] has a wavelength of 0.128 nm and pulse duration of 10 fs. Comparing the CR output at harmonic 20 with the seed laser 2 of eehg1, there is a factor of 0.5 shortening in the pulse duration; similarly, there is a factor of 0.4 shortening in the eehg2 beamline at harmonic 100. Compared with the CR output of radiator 2, the unseeded part has an intensity similar to the background level.

### 3.2. Case 2: SASE

**Figure 10:**
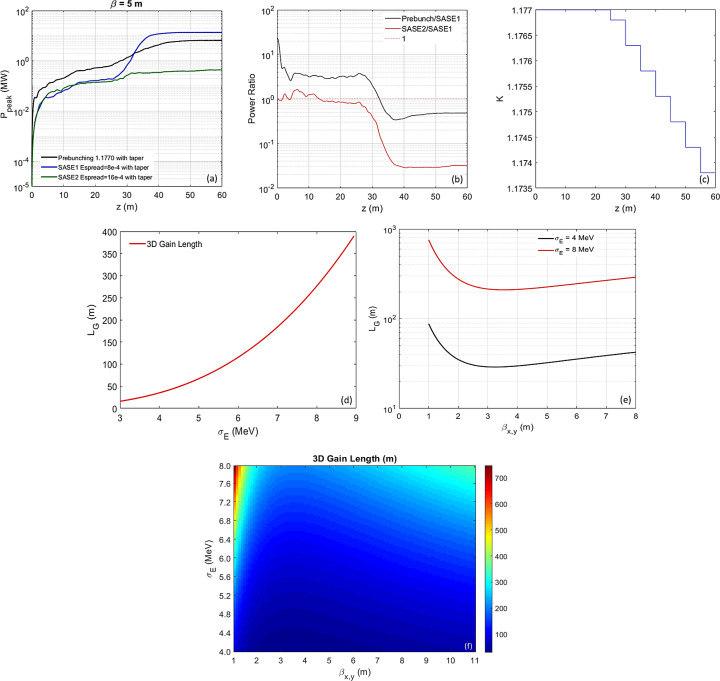
(*a*) Regarding the cascaded EEHG with harmonic 2000, averaged β_*x*,*y*_ = 5 m and *I*_peak_ = 200 A, the radiator-2 is set to lase in the fundamental with an undulator period of 10 mm; the peak power as a function of the undulator distance is shown for the optimal detuning case with *K*_u,helical_ = 1.1770 with additionally optimized tapering (black), SASE1 with optimized tapering and equilibrium energy spread 8 × 10^−4^ (blue), and SASE2 with optimized tapering and doubled energy spread 1.6 × 10^−3^ (green). (*b*) Ratio of CR peak powers for two cases – prebunching with SASE1 ( 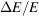 = 8 × 10^−4^) (black) and SASE2 ( 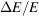 = 1.6 × 10^−3^) with SASE1 (red), as a function of undulator distance. (*c*) The tapering (*k* versus *z*) is plotted. (*d*) 3D gain length (*L*_G_) as a function of the RMS energy spread in units of MeV. (*e*) *L*_G_ as a function of β_*x*,*y*_ with two different RMS energy spreads, 4 MeV (black) and 8 MeV (red). (*f*) Contour of 3D gain length as functions of β_*x*,*y*_ in *x* and RMS energy spread σ_E_ in *y*.

Since it was numerically confirmed in Section 3.1 that the prebunching performs in a similar manner while β_*x*,*y*_ varies in the range 2–5 m, we now fix β_*x*,*y*_ to a more realistic value of 5 m and extend the undulator length to 60 m. With optimized detuning [black curve in Fig. 8 ▸(*d*)], a peak power of 3 MW can be achieved with an undulator distance of 38 m; with additional optimized tapering starting from 25 m [Fig. 10 ▸(*c*)], the CR peak power can reach more than 6.5 MW with an undulator distance of 60 m [black curve in Fig. 10 ▸(*a*)]. Furthermore, we compare the optimized detuning [black curve in Fig. 8 ▸(*d*)] with additional tapering to SASE with two different relative electron beam energy spreads, one with an equilibrium value of 8 × 10^−4^ [named SASE1, blue curve in Fig. 10 ▸(*a*)] and the other with a doubled energy spread of 1.6 × 10^−3^ [named SASE2, green curve in Fig. 10 ▸(*a*)] resembling the energy modulated prebunching case. The SASE schemes could be implemented in a ring with long straight sections through a bypass line that hosts the long undulator. The electron bunch is kicked into the bypass line to generate a FEL with a fast kicker and allowed to damp down to the equilibrium distribution afterwards. It is evident that there is a significant benefit from the prebunching – a significant higher CR peak power, when the undulator distance *L*_u_ is small, especially within a few metres. However, when *L*_u_ > 30 m, the FEL gain is dominated by the initial energy spread, and the ratio of peak powers between the prebunching and SASE1 with 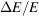 = 8 × 10^−4^ becomes less than 1, as shown in Fig. 10 ▸(*b*). Comparing these two SASE cases, SASE1 with a small energy spread has a much higher CR peak power; moreover, this difference increases with undulator distance. The ratio of peak powers between SASE2 and SASE1 stays close to 1 when *L*_u_ < 30, and becomes much smaller than 1 with increasing *L*_u_, shown as the red curve in Fig. 10 ▸(*b*). Beam heating has been kept below 50% at the exit of the undulator for both the prebunching and SASE1; such a heating effect can be completely damped down to the equilibrium energy spread within one to two radiation damping times (∼50–100 ms).

When the beta functions have their nominal design values and the allowable distance of the radiator is large (>30 m), the SASE FEL process will go through the following processes: (i) startup from shot noise induced by the discrete nature of electrons and (ii) exponential growth. The 3D gain length *L*_G_ as a function the RMS electron beam energy spread is estimated via Ming Xie’s fitted formula (Xie, 1995 ▸) and shown in Fig. 10 ▸(*d*); *L*_G_ as a function of β_*x*,*y*_ is shown in Fig. 10 ▸(*e*) with two different RMS energy spreads, 4 MeV (black) and 8 MeV (red). It is evident that the FEL gain process is dominated by the electron beam energy spread, rather than the beta function, since its dependence on the beta function is much weaker than the energy spread, shown as the 3D gain length contour as a function of β_*x*,*y*_ (*x*-axis) and σ_E_ (*y*-axis) in Fig. 10 ▸(*f*).

### 3.3. Comparison of EEHG with SASE

So far we have studied two cases: case 1 – prebunching with the undulator length limited to 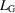 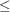 30–40 m; and case 2 – SASE with the undulator distance up to 60 m. Compared with SASE, EEHG is quite different because of the unique combination of large prebunching and no radiation field initially. Besides, in EEHG there is bunching at multiple neighboring wavelengths, with many micro-bunches somewhat displaced from where they ideally should be. Thus, one cannot assume that the initial EEHG prebunching would be anything like the bunching after amplification. It is reasonable for us to treat the initial bunching as fixed to generate the CR radiation, and separately to consider a ‘new’ bunching generated by interactions with the radiation and exponentially growing in the long undulator. To take a specific case into account, shown as the black curve in Fig. 10 ▸(*a*), the initial prebunching provides the main portion of the total radiation (in the first few metres where the FEL process is dominated by the prebunching induced CR, far superior than SASE); but there is also a moderate exponential gain increasing with the undulator length, eventually becoming the dominant part of the total radiation when the undulator distance *L*_u_ ≥ *z*_th_ (≃ 60 m). Case 1 prefers a small β_*x*,*y*_, thus small transverse beam sizes, and the FEL process is dominated by the prebunching-induced CR process. Since case 2 features a long undulator (*L*_u_ ≃ 60 m), the FEL process is dominated by the initial energy spread. 3D gain length increases rapidly with increasing initial energy spread. A nominal SASE process starting with the equilibrium energy spread in conjunction with the tapering can yield significantly higher peak powers [blue curve in Fig. 10 ▸(*a*)]. One can effectively utilize tapering to extract more energy from the electron beam to the radiation field. This can be understood via equation (7),

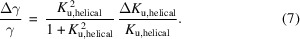

**Figure 11:**
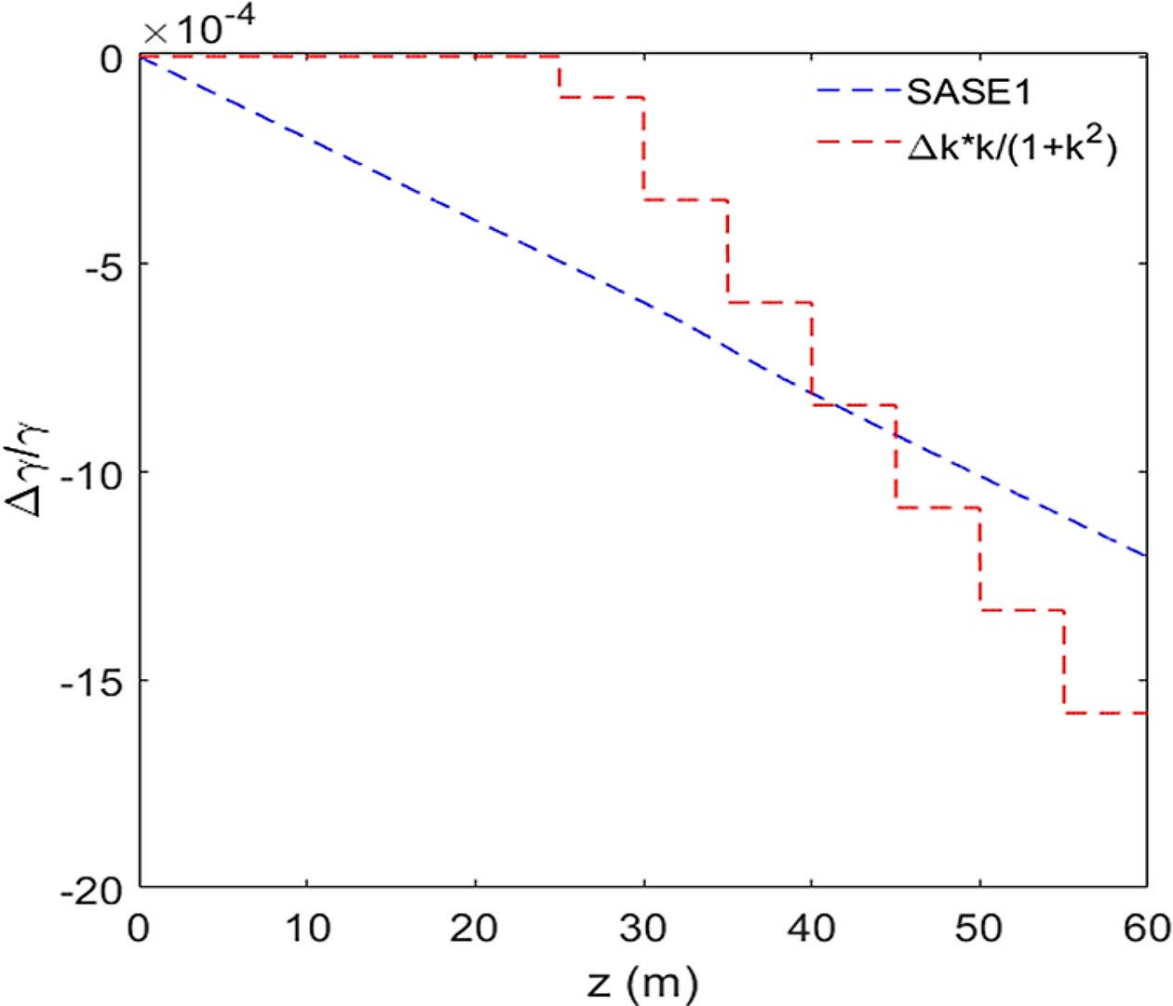
The normalized electron beam centroid energy as a function of the radiator distance is plotted as the blue curve. The right side of equation (7) is plotted as the red curve.

Regarding SASE1, the left and right sides of equation (8) are plotted as the blue and red curves in Fig. 11 ▸, respectively; at *z* = 60 m, they agree reasonably well.

For these two cases, where there is space limitation, prebunching should be the optimal choice; if there is no space limitation, SASE can be applied to achieve the highest photon flux.

## 4. Photon properties

**Figure 12:**
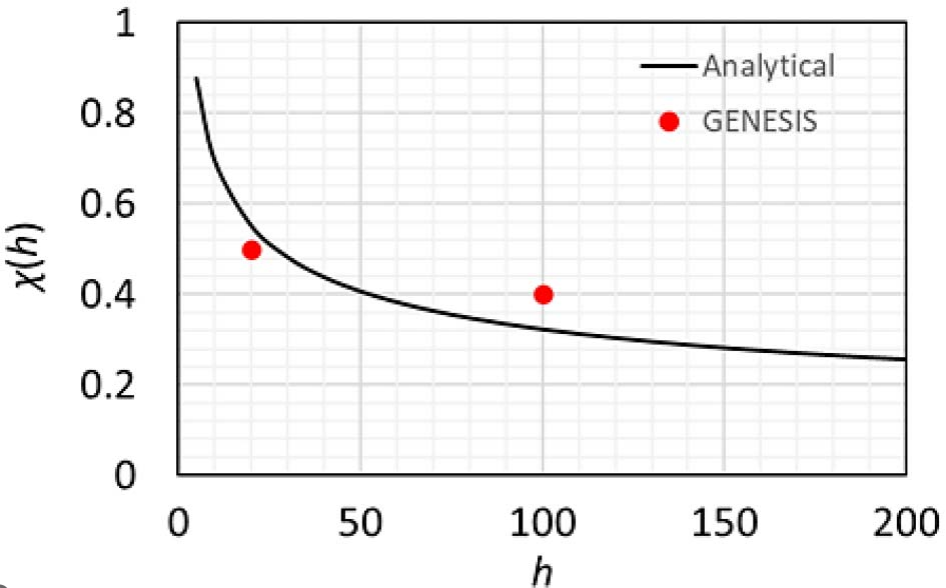
With the assumption that seed lasers 1 and 2 have similar pulse durations, the ratio of pulse durations of CR and seed laser 2 (χ) as a function of the harmonic *h* is estimated via equation (8) (black curve) and confirmed by *GENESIS* simulation at harmonics 20 and 100 (red dots).

We assume that the longitudinal profiles of seed lasers 1 and 2 are Gaussian with a similar full width at half-maximum. The final pulse duration will be shorter than the length of seed laser 2 (σ_t2_) by a factor of χ [see equation (8)] (Raimondi *et al.*, 2023 ▸),

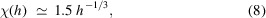

where *h* is the harmonic number of the second beamline of the cascaded EEHG, as shown in Fig. 12 ▸. The CR output is estimated for the hard X-ray wavelength of 0.128 nm, including the peak power, number of photons per pulse and RMS spectral bandwidth for two different pulse durations in RMS (σ_*r*,τ_): 0.212 ps and 0.425 ps. For *h*_2_ = 100, χ is equal to 0.324. The corresponding RMS pulse durations of seed laser 2 need to be 3 × σ_r,τ_, *i.e.* 0.64 ps and 1.23 ps, respectively. For each case, these output properties are estimated in two different DL-SLSs, the NSLS-II upgrade with *L*_u_ = 4 m and SDLS with *L*_u_ = 40 m, and β_*x*,*y*_ = 2 m to guarantee the 3D full coherence [Fig. 8 ▸(*b*)], as shown in Table 4 ▸.

For the case of the SDLS lattice with an undulator distance of 40 m and RMS pulse duration of 0.425 ps, the maximum number of photons per pulse is 2.4 × 10^10^ with λ_r_ = 0.128 nm. To overcome the radiation damping time via modulating different portions of the electron bunch iteratively, the repetition rate can be increased to 1 kHz (Yang *et al.*, 2022*a* ▸,*b* ▸); hence, the corresponding spectral flux is 1.7 × 10^21^ photons s^−1^ (0.1% bandwidth)^−1^, which is more than a million times higher than the current brightest source IVU20 [∼2 × 10^14^ photons s^−1^ (0.1% bandwidth)^−1^] at NSLS-II (Dierker, 2007 ▸; Tanabe, 2007 ▸; Chubar *et al.*, 2013 ▸).

There are two important issues which ultimately limit the averaged CR power (Yang *et al.*, 2022*b* ▸) when one considers implementing the cascaded EEHG scheme in a SLS. First, the average power will depend on the repetition rate of the seed laser, especially when the required seed laser pulse energy is quite large. Second, the beam heating that is caused by the repeated interaction with the seed laser pulse needs to be well compensated by the radiation damping.

To address the first issue, we study the second cascaded EEHG beamline which is the worst-case scenario when the harmonic equals 100 and 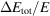 is about 0.4%. This case requires a seed laser pulse energy up to a few hundred µJ { 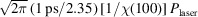 ≃ 160 µJ} to produce a CR pulse duration of 1 ps FWHM. This large energy needed for each seed laser pulse could ultimately limit the repetition rate to 1 kHz with the commercially available laser technologies, assuming 10% efficiency for third-harmonic generation from a Ti:sapphire laser (Del Mar Photonics, https://www.dmphotonics.com/index.htm; MKS Instruments Light & Motion Division: Spectra-Physics, https://www.spectra-physics.com). To explore the repetition rate limited by the beam heating induced by the energy modulation, we performed a particle-tracking simulation in our early studies for the case with 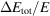 = 0.004 based on the APS-U lattice (Yang *et al.*, 2022*b* ▸; APS, 2019 ▸; Borland, 2000 ▸; Terebilo, 2001 ▸). Quantum excitation and radiation damping are turned on in the tracking setup. The equilibrium distribution without any energy modulation is used as the initial distribution; then, a laser-induced energy modulation is added to the center part of the distribution. The tracking simulation confirmed that (i) the increase in RMS energy spread due to the modulation is nearly damped out after a radiation damping time (20 ms); (ii) it takes up to one to two horizontal damping times (7–14 ms) for the energy-modulation-induced beam heating in the horizontal emittance to be damped out (Yang *et al.*, 2022*b* ▸). As a result, modulating different parts of an electron bunch in conjunction with operating in the multi-bunch mode will allow an increase in the repetition rate, toward the ultimate limit set by the required seed laser power, ∼1 kHz.

## 5. Method

To extend SR-based FELs toward the tender and hard X-ray wavelengths, we choose the cascaded EEHG to fulfill the required high harmonic from 500 to 2000 with a seed laser wavelength of 256 nm. To overcome the space limitation as well as mitigate the ISR effect, one can apply a compact design with the stage-1 modulator in common for cascaded EEHG beamlines, as shown in Fig. 1 ▸ for the NSLS-II upgrade and Fig. 6 ▸ for the SDLS.

Regarding the first part of the cascaded EEHG, the EEHG approach is not the only option. Since the harmonic is so low, *i.e.* *h*_1_ = 20, there are a few other choices based on the steady-state microbunching (SSMB) method, which can be quite effective when *h*_1_ ≤ 20 (Lu *et al.*, 2022 ▸), *e.g.* angular-dispersion-induced microbunching (Feng & Zhao, 2017 ▸; Li *et al.*, 2020 ▸), obliquely incident seed lasers (Wang *et al.*, 2019*a* ▸,*b* ▸; Li *et al.*, 2021 ▸) and TEM01 mode laser bunching schemes (Zholents & Zolotorev, 2008 ▸; Xiang & Wan, 2010 ▸). Comparing EEHG with other SSMB-based options, one can take full advantage of utilizing different numbers of BM sections as the first chicanes for both EEHG beamlines; thus, fewer lattice changes are needed.

## 6. Conclusion

There is a worldwide trend to combine SLS and FEL sources on the same site, *e.g.* SSRL and LCLS (USA), PETRA-III and EuXFEL (Germany), SPring-8 and SACLA (Japan), ELETTRA and FERMI (Italy). To expand the capabilities of the NSLS-II upgrade and a potential future DL-SLS in the PEP tunnel, we are considering a few FEL options using a low-emittance electron beam of the NSLS-II upgrade and SDLS and synergetic with SR operations. The cascaded EEHG seeding option has been numerically demonstrated with the capability of generating very narrow bandwidths and extremely high brightness, realized by diffraction-limited short pulses in transverse planes and Fourier-limited bandwidth in the tender to hard X-ray spectrum (Li *et al.*, 2020 ▸, 2021 ▸; Wang *et al.*, 2019*a* ▸,*b* ▸; Zholents & Zolotorev, 2008 ▸; Xiang & Wan, 2010 ▸; Nuhn *et al.*, 1992 ▸; Zhao, 2010 ▸; Mitri & Cornacchia, 2015 ▸). Regarding the fourth-generation DL-SLSs, momentum compactions are significantly smaller; thus, to cover the hard X-ray spectrum with up to 10 keV photon energy, we propose a specific design when there is space limitation – sharing the common stage-1 modulator via twin-pulse seeding of the same electron bunch scheme (Yang *et al.*, 2022*a* ▸,*b* ▸, 2023 ▸) or two longitudinally delay-adjustable electron bunches with a seed-laser wavelength of 256 nm, then cascading two pairs of stage-2 modulators and radiators, where the output of radiator 1 is used as an input seed to the second stage-2 modulator in the cascaded EEHG. This approach can generate a significant prebunching with up to 2000 harmonics based on the NSLS-II upgrade lattice. Furthermore, we show that this standardized design of cascaded EEHG is applicable to almost all future DL-SLSs with significant benefits.

Thanks to a much higher electron beam energy and ultra-low emittances, DL-SLSs with long straight sections, such as PETRA-IV and SDLS, can take the full potential via an ideal design of the cascaded EEHG. Moreover, we compared two cases with different undulator distances – one has a space limitation of a few metres and the other can accommodate 10 to 100 m of undulator. In the first case with a strict space limitation, one can benefit significantly from the prebunching, whereas in the second case, without space limitation, the FEL gain is dominated by the electron beam energy spread. One can consider other options, *e.g.* SASE and X-ray FEL oscillator (XFELO). Among those cases, where there is a space limitation, prebunching should be the best choice; if there is no space limitation, SASE or XFELO can be applied depending on user preference – SASE can provide a high photon flux and XFELs could offer the narrowest spectral bandwidth. Detailed comparisons of these available schemes will be our future studies.

## Figures and Tables

**Table 1:** List of lattice parameters, including beam energy, circumference, beta function, peak current, bunch length and momentum compaction for the NSLS-II and its future upgrade of the fourth-generation SLS and a recent storage ring lattice design in the PEP tunnel named SDLS (Raimondi *et al.*, 2023 ▸) α_c_ and *R*_56_ are the momentum compactions of a SR lattice and one BM section, respectively. The peak current (*I*_peak_) mainly depends on the specific mode of operation. We assume *I*_peak_ = 300 A for the NSLS-II upgrade and *I*_peak_ = 150–200 A for the SDLS in simulations. Regarding the peak current *I* of the NSLS-II upgrade, the final CR power can be scaled quadratically by the factor 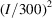 (Yang *et al.*, 2022*a* ▸,*b* ▸) since this configuration does assume a peak current of 300 A, which is significantly above the value limited by the intra-beam scattering (IBS); but in the tender X-ray regime such a large current would not be required.

	Energy (GeV)	Circumference (m)	Emittance (nm)	β_*x*,avg_ (m)	β_*y*,avg_ (m)	*I*_peak_ (A)	σ_t_ (ps)	α_c_	*R*_56_ (mm)	σ_E_/*E*	No. of cells	ID length (m)
NSLS-II	3.00	791.96	1.00	3.29	3.05	100–300	20.0	3.63 × 10^−4^	9.6	1.0 × 10^−3^	30	5.00
NSLS-IIU	3.00	791.96	0.03	1.90	1.80	100–300	20.0	5.12 × 10^−5^	1.4	8.0 × 10^−4^	30	6.00
SDLS	5.00	2189.80	0.02	le 5	le 5	150-200	3.7	2.62 × 10^−5^	9.6	8.0 × 10^−4^	6	120.00

**Table 2:** Optimized cascaded EEHG parameters for the first and second beamlines, eehg1 and eehg2

	A1	*P*_laser1_ (MW)	λ_mod1_ (cm)	*L*_mod1_ (m)	*B*_peak_ (T)	*R*_1_ (mm)	λ_1_ (nm)	*A*_2_	*P*_laser2_ (MW)	λ_mod2_ (cm)	*L*_mod2_ (m)	*B*_peak_ (T)	*R*_2_ (µm)	λ_2_ (nm)	Δ*E*_tot_ (MeV)	*b*_*n*_ (%)	λ_rad1_ (cm)	λ_rad2_ (cm)
eehg1	1.30	0.60	20.00	2.00	0.71	4.20	256	0.25	0.16	20.0	2.0	0.71	−212.1	256	3.3	4.9	6.4	
eehg2	1.30	12.46	20.00	2.00	0.71	14.00	256	0.38	6.90	6.4	2.0	0.85	−7.0	12.8	3.3	4.6		1.0

**Table 3:** The optimized cascaded EEHG parameters for the first and second beamlines, named eehg1 and eehg2

	*A*_1_	*P*_laser1_ (MW)	λ_mod1_ (cm)	*L*_mod1_ (m)	*B*_peak_ (T)	*R*_1_ (mm)	λ_1_ (nm)	*A*_2_	*P*_laser2_ (MW)	λ_mod2_ (cm)	*L*_mod2_ (m)	*B*_peak_ (T)	*R*_2_ (µm)	λ_2_ (nm)	Δ*E*_tot_ (MeV)	*b*_*n*_ (%)	λ_rad1_ (cm)	λ_rad2_ (cm)
eehg1	1.30	2.90	20.00	2.00	1.18	9.60	256	0.11	0.15	20.0	2.0	1.18	−482.1	256	5.4	8.1	6.4	
eehg2	1.30	58.60	20.00	2.00	1.18	9.60	256	0.55	71.50	6.4	2.0	1.40	−4.8	12.8	5.7	4.7		1.0

**Table 4:** CR at a photon energy of 10 keV, including the peak power, number of photon pulses and RMS spectral bandwidth in two different pulse durations in RMS: 0.212 ps and 0.425 ps For each case, the output properties are calculated at two different DL-SLSs: the NSLS-II upgrade with *L*_u_ = 4 m and SDLS with *L*_u_ = 40 m.

	*P*_pk_ (MW) at *E*_ph_ = 10 keV		Photons pulse^−1^ at *E*_ph_ = 10 keV		RMS bandwidth		Photons s^−1^ at 1 kHz		Spectral flux at 1 kHz	
*T*_RMS_ (ps)	*L*_NSLSIIU_ = 4 m	*L*_SDLS_ = 40 m	*L*_NSLSIIU_ = 4 m	*L*_SDLS_ = 40 m	*L*_NSLSIIU_ = 4 m	*L*_SDLS_ = 40 m	*L*_NSLSIIU_ = 4 m	*L*_SDLS_ = 40 m	*L*_NSLSIIU_ = 4 m	*L*_SDLS_ = 40 m
0.212	0.17	3.5	5.70 × 10^8^	1.2 × 10^10^	4.3 × 10^−5^	2.6 × 10^−5^	5.70 × 10^11^	1.2 × 10^13^	1.3 × 10^19^	4.4 × 10^20^
0.425			1.14 × 10^9^	2.4 × 10^10^	2.1 × 10^−5^	1.3 × 10^−5^	1.14 × 10^12^	2.4 × 10^13^	5.43 × 10^19^	1.7 × 10^21^
